# A complex network of QTL for thousand-kernel weight in the rye genome

**DOI:** 10.1007/s13353-020-00559-3

**Published:** 2020-04-30

**Authors:** Piotr Masojć, Piotr Kruszona, Anna Bienias, Paweł Milczarski

**Affiliations:** Department of Plant Genetics, Breeding and Biotechnology, West Pomeranian University of Technology in Szczecin, Słowackiego 17, 71-434 Szczecin, Poland

**Keywords:** *Secale cereale* L., DArT markers, High-density map, QTL mapping, Thousand-kernel weight, Two-loci interactions

## Abstract

Here, QTL mapping for thousand-kernel weight carried out within a 541 × Ot1-3 population of recombinant inbred lines using high-density DArT-based map and three methods (single-marker analysis with *F* parametric test, marker analysis with the Kruskal–Wallis K* nonparametric test, and the recently developed analysis named genes interaction assorting by divergent selection with χ^2^ test) revealed 28 QTL distributed over all seven rye chromosomes. The first two methods showed a high level of consistency in QTL detection. Each of 13 QTL revealed in the course of gene interaction assorting by divergent selection analysis coincided with those detected by the two other methods, confirming the reliability of the new approach to QTL mapping. Its unique feature of discriminating QTL classes might help in selecting positively acting QTL and alleles for marker-assisted selection. Also, interaction among seven QTL for thousand-kernel weight was analyzed using gene interaction assorting by the divergent selection method. Pairs of QTL showed a predominantly additive relationship, but epistatic and complementary types of two-loci interactions were also revealed.

**Electronic supplementary material:**

The online version of this article (10.1007/s13353-020-00559-3) contains supplementary material, which is available to authorized users.

## Introduction

A complex genetic background of thousand-kernel weight (TKW), one of the most important components of cereal yield, has been detected in rye (Milczarski and Masojć 2003; Falke et al. 2009; Miedaner et al. 2012; Myśków et al. 2014; Masojć et al. 2017; Hackauf et al. 2017), wheat (Börner et al. 2002; Groos et al. 2003; Cuthbert et al. 2008; Sun et al. 2009; Ramya et al. 2010; Cui et al. 2011; Mohler et al. 2016), and barley (Bezant et al. 1997; Teulat et al. 2001; Tsilo et al. 2010) by means of QTL mapping. Several QTL for TKW in rye were found on each of the seven chromosomes in various map intervals (Milczarski and Masojć 2003; Falke et al. 2009; Miedaner et al. 2012; Myśków et al. 2014; Masojć et al. 2017; Hackauf et al. 2017).

Because a single QTL of the predominantly strong effect on TKW has not been detected so far in rye, the most reliable strategy of selection is pyramiding positively acting alleles from several effective QTL in one variety. Identification of three or four loci genotypes stabilizing TKW on the highest possible level is a challenging task for rye geneticists. One of the questions which should be addressed in the course of these investigations is the role of genetic interactions that might enhance or reduce particular alleles effects (Tranquilli and Dubcovsky 2000; Long et al. 2008; Deng et al. 2014).

Genetic analysis carried out within-population tails suggests a substantial role of two-loci interactions in controlling phenotypic variation in the rye (Masojć et al. 2016). This hypothesis is based on frequent detection of QTL representing R and E classes showing alleles-trait association only in one of the two population tails (i.e., that representing desirable phenotype (R class loci) or the opposite, gathering lines of negative characteristics (E class locus)). As shown in a recently developed genetic model (Masojć et al. 2016), QTL of R and E classes reflect epistatic interaction with QTL of class D (directional), revealing alleles-trait association within both population tails. The method of QTL classification is known as genes interaction assorting by divergent selection (GIABDS) (Masojć et al. 2016) and represents further development of bidirectional selective genotyping (BSG), used by many authors for QTL identification (Gallais et al. 2007; Navabi et al. 2009; Sun et al. 2010; Farkhari et al. 2013; Myśków and Stojałowski 2016). Both methods apply the divergent selection to generate two subpopulations with contrasting phenotypes and look for significant differences in allele frequencies to disclose QTL. GIABDS is carried out within bi-parental populations of recombinant inbred lines where QTL detection relies on finding a significant deviation from the expected 1:1 allelic segregation ratio among selected lines with extreme phenotypes. So far, studies using GIABDS method have allowed us to characterize the genetic architectures of 11 quantitative traits in rye (Masojć et al. 2009, 2011, 2017). Results of GIABDS were shown to comply with those of classic QTL mapping on examples of pre-harvest sprouting and alpha-amylase activity in rye (Masojć and Milczarski 2009; Masojć et al. 2009, 2011). Revealing QTL classes by GIABDS allowed us to select those having a significant impact on desirable traits since only the D and R or R* classes are useful for trait improvement. QTL of class E and E* are not effective as selection tools and can be omitted. This property of GIABDS analysis can significantly reduce the number of molecular marker loci planned to be developed for selection aims.

This paper is aimed at the characterization of QTL for TKW in the 541 × Ot1-3 mapping population of rye using three methods of QTL detection (GIABDS, SMA (single-marker analysis) and Kruskal–Wallis (K–W) test)). This is also the first attempt to study two-loci interactions using a recently developed model for genetic analysis within-population tails.

## Materials and methods

### Plant materials

The 541 × Ot1-3 mapping population of rye consisting of 144 recombinant inbred lines (RILs) representing *F*_> 11_ generations was propagated in 2015–2017 on the experimental field of West Pomeranian University of Technology, Szczecin, Poland. Each RIL was grown in a 1-m row with 20 cm interspace. Individual spikes were bagged before the pollination period to avoid outcrossing. Mature spikes were hand-threshed, and grain from 6 to 10 plants per line was collected and stored at room temperature in paper bags. TKW was evaluated using an automatic seed counter and electronic weight with an accuracy of 0.1 g. The two subpopulations of RILs, first representing the lowest and second the highest TKW values, were selected from the lower and upper population tails, respectively. Selected subpopulations consisted of 24 (upper tail) and 25 (lower tail) RILs. The averaged data was used.

### QTL mapping

RIL genotypes were derived from marker segregation data of the high-density DArT-based genetic map of rye developed on the 541 × Ot1-3 mapping population by Milczarski et al. (2011). Classic QTL mapping performed through single marker analysis (SMA) using Windows QTL Cartographer 2.5 (Wang et al. 2012), K–W using MapQTL 5 (Van Ooijen 2004), and GIABDS, a method based on a recently developed genetic model (Masojć et al. 2016), were applied for QTL mapping. QTL were revealed in map positions where at least three consecutive markers showed significant association with TKW. QTL was denoted using trait symbol (TKW) followed by chromosome localization and its consecutive number on the chromosome map. Statistical analyses were based on likelihood ratio (LR) and *F* tests (*p* < 0.05) in SMA and on nonparametric Kruskal–Wallis K* test (*p* < 0.05) (Lehmann 1975) as well as on χ^2^ test (*p* < 0.05) examining the significance of distortion from the 1:1 segregation ratio of AA and BB genotypes within each of the two population tails in GIABDS method. Effectiveness of particular QTL in TKW control was characterized by the coefficient of determination (*R*^2^) in SMA and by the difference in genotypic values of AA (parental line 541) and BB (parental line Ot1-3) genotypes in GIABDS method. The genotypic value was assessed as a mean of phenotypic values for 45–56 RILs representing the same single-locus homozygote. Genotypic values were presented in percent, where 100% value was attributed to the highest level of TKW, detected within the mapping population (32.0 g). The only QTL with genotypic values differing considerably (more than 5.0%) were included in the analysis of two-loci interactions.

### GIABADS method

QTL were classified according to the system developed earlier (Masojć et al. 2009, 2011, and 2017). Significant distortion from the expected 1:1 segregation found within both selected subpopulations, with an overrepresentation of different alleles identified QTL of class D (directional). If segregation distortion was detected only within the subpopulation of high TKW values and frequencies of genotypes were close to 1:1 ratio within the opposite subpopulation, the R class, was assigned to QTL. QTL of class E was reported when segregation distortion was proved within a subpopulation of low TKW and a 1:1 ratio of genotypes frequencies pertained with high TKW group. The R* class was distinguished for QTL showing segregation distortion (AA genotype in excess) within the upper tail and segregation ratio close to 2:1 (BB genotype in excess) within the lower tail. According to the genetic model (Masojć et al. 2016), the class of the QTL indicates its relationship with other QTL.

The relationship between two QTL controlling TKW was defined by comparing the genotypic values of the four double homozygotes detected within the mapping population of RILs. The genotypic value for two-loci genotype was calculated as a mean of 20–35 RILs representing one of the double homozygotes (AA;AA, AA;BB, BB;AA, or BB;BB).

Differences between genotypic values of the two double homozygotes lower than 5% (below a 4.5% threshold) were assumed to be insignificant or ineffective for divergent selection. Distribution of significant and insignificant genotypic differences between four double homozygotes was related to the type of two-loci interaction and alleles distribution in selected subpopulations, as shown in the model (Masojć et al. 2016). An additive relationship between two QTL (D-D type of interaction) was declared when significant allelic effects found at individual loci were also detectable on the level of two-loci genotypes (genotypic differences above a 4.5% threshold). Insignificant difference between genotypic values of AA;AA and AA;BB double homozygotes indicated the D-E type of interaction, while the insignificant difference between genotypic values of BB;AA and BB;BB homozygotes suggested the D-R type of interaction. Both D-E and D-R types of interaction were considered as an epistatic relationship, where the presence of one allele at the D class locus repressed differentiation of allelic effects at the second locus. The E*-E* type of interaction was demonstrated by similar genotypic values of three double homozygotes: AA;AA, AA;BB, and BB;AA and substantially lower value of the BB;BB genotype. This type of interaction corresponded to the complementary relationship between two QTL, where only in the presence of B alleles at both loci a strong negative effect on the trait value was observed. A characteristic feature of the R*-R* type of interaction was similar genotypic values of the three double homozygotes, i.e., AA;BB, BB;AA, BB;BB, and significantly higher genotypic value of homozygote AA;AA. This interaction also represented the complementary relationship between QTL, where the strong positive effect on the studied trait could be observed only in the presence of A alleles at both loci.

## Results

### Characterization of the mapping population concerning TKW variation

**Fig. 1:**
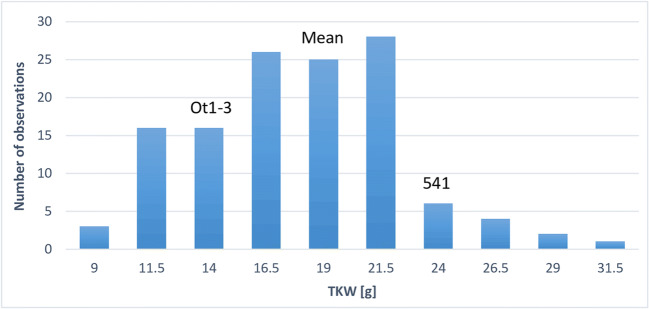
Distribution of thousand-kernel weight (TKW) in recombinant inbred lines of the 541 × Ot1-3 mapping population of rye in 2017

TKW showed a wide variation range (9.2–32.0 g) within the 541 × Ot1-3 mapping population in 2017 (Fig. 1), substantially exceeding that represented by parental lines: Ot1-3 of genotype BB (15.4 g) and 541 of genotype AA (24.3 g). The population mean was 19.3 g (60.4%), and those for selected subpopulations with the lowest and the highest TKW were 12.5 g (39.1%) and 25.9 g (80.9%), respectively. TKW values in a subpopulation of 24 RILs representing lower tail ranged from 9.2 to 14.9 g and those in a subpopulation of 25 RILs representing upper tail—from 21.7 to 32.2 g (S1 File).

### QTL mapping

**Fig. 2:**
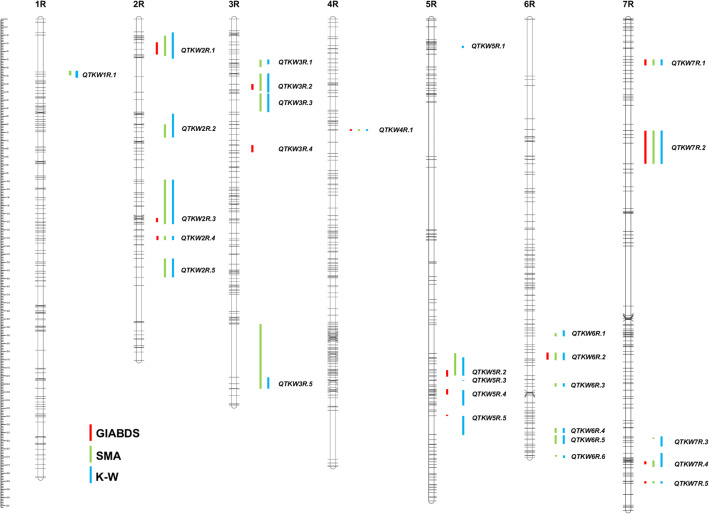
QTL mapping for thousand-kernel weight within 541 × Ot1-3 population of recombinant inbred lines of rye using three methods: genes interaction assorting by divergent selection (GIABDS), single marker analysis (SMA), and Kruskal–Wallis test (K–W). DArT-based high-density genetic map developed by Milczarski et al. (2011) was used

A total of 28 of QTL for thousand kernel weight were detected using three methods of QTL mapping (Fig. 2). The highest number of QTL was revealed by the K–W method (27). Only four QTL out of 27 found using the K–W test was not confirmed using SMA. QTL range was very similar for SMA and K–W in the majority of loci. Thus, both methods gave highly similar results of TKW architecture. GIABDS elucidated 13 QTL coinciding with those found by the two remaining methods and one specific locus on chromosome 3R (*QTKW3R.4*). Usually, the range of QTL detected by GIABDS was narrow in respect to that displayed by SMA and K–W, but in a few cases (*QTKW2R.4*, *QTKW4R.1*, *QTKW6R.2*, *QTKW7R.1*, *QTKW7R.2*, and *QTKW7R.5*) it was similar to those determined by classic methods. QTL were not evenly distributed along rye chromosomes. The highest number of QTL for TKW contained chromosomes 6R (6), 2R (5), 3R(5), and 7R (5). Single QTL were found on chromosomes 1R and 4R. Statistic tests for QTL significance are shown in Table 1 with supplementary File S1 (GIABDS method) and Table 2. The highly distorted segregation ratio of AA vs. BB genotypes was found in both population tails for *QTKW7R.5* (QTL of class D), only within a lower population tail for QTKW2R.1 (class E) or only within upper population tail for the remaining 12 QTL (class R or R*). According to the SMA and K–W methods, the highest significance level was found for QTL: *QTKW2R.1*, *QTKW2R.3*, *QTKW2R.4*, *QTKW2R.5*, *QTKW7R.4*, and *QTKW7R.5* (Table 2). The coefficient of determination (*R*^2^) overcame the 7.0% value only for QTL: *QTKW2R.1*, *QTKW2R.2*, *QTKW2R.3*, *QTKW2R.4*, *QTKW2R.5*, *QTKW 7R.4*, and *QTKW7R.5*. The remaining QTL have rather low *R*^2^ values (2.5–6.5%), which shows that they can exert stronger effects on TKW only by cumulative action.

**Table 1:** Characterization of QTL for thousand-kernel weight (TKW) in rye detected within the 541 × Ot1-3 mapping population of recombinant inbred lines, using the method of genes interaction assorting by divergent selection (GIABDS)

QTL	Map position (cM)	Flanking markers	Ratio of AA and BB genotypes within selected subpopulation with the highest TKW values	χ^2^ (1:1)	Ratio of AA and BB genotypes within selected subpopulation with the lowest TKW values	χ^2^ (1:1)	QTL class
*QTKW2R.1*	14.7	XrPt389332	10:14	0.67	6:19	6.76**	E
	22.5	XrPt116273					
*QTKW2R.3*	122.8	XrPt345057	18:6	6.00*	10:15	1.00	R
	125.2	XrPt508720					
*QTKW2R.4*	133.6	XrPt400997	19:5	8.17**	11:14	0.36	R
	136.0	XrPt399895					
*QTKW3R.2*	40.2	XrPt349181	21:3	13.5***	10:15	1.00	R
	43.5	XrPt348182					
*QTKW3R.4*	77.7	XrPt399830	19:5	8.17**	12:13	0.04	R
	82.2	XrPt505832					
*QTKW4R.1*	68.2	XrPt508070	20:4	10.67**	13:12	0.04	R
	68.4	XrPt402117					
*QTKW5R.2*	215.9	XrPt348431	19:5	8.17**	9:16	1.96	R
	220.3	XrPt505467					
*QTKW5R.4*	228.1	XrPt401944	18:6	6.00*	11:14	0.36	R
	231.2	XrPt346964					
*QTKW5R.5*	244.3	XrPt411479	18:6	6.00*	11:14	0.36	R
	244.3	XrPt346862					
*QTKW6R.2*	205.4	XrPt506201	18:6	6.00*	12:13	0.04	R
	209.9	XrPt400819					
*QTKW7R.1*	25.1	XrPt506468	18:6	6.00*	8:17	3.24	R*
	26.6	XrPt505798					
*QTKW7R.2*	68.7	XrPt410779	19:5	8.17**	8:17	3.24	R*
	89.2	Xopq4L578					
*QTKW7R.4*	272.3	XrPt343849	19:5	8.17**	9:16	1.96	R
	274.2	XrPt389812					
*QTKW7R.5*	284.6	XrPt346921	18:6	6.00*	6:19	6.76*	D
	285.9	XrPt509722					

*, **, *** significant at *p* < 0.05, *p* < 0.01, and *p* < 0.001, respectively

**Table 2:** Confirmation of QTL for thousand-kernel weight, detected using Genes Interaction Assorting by Divergent Selection (GIABDS) method, through classic QTL analysis: SMA (Single Marker Analysis) and K–W (Kruskal–Wallis test) of the entire 541 × Ot1-3 mapping population of recombinant inbred lines in 2017

	GIABDS	Single Marker Analysis				Kruskal–Wallis		
QTL	Position^a^	Position^a^	LR	Significance at *p*<	*R*^2^ (%)	Position^a^	K^*^	Significance at *p*<
*QTKW1R.1*		4–10	7.08	0.01	6.40	8–12	8.40	0.005
*QTKW2R.1*	14–18	15–20	9.82	0.01	7.65	2–21	10.80	0.001
*QTKW2R.2*		33–37	7.04	0.01	7.60	28–38	6.71	0.01
*QTKW2R.3*	69–77	55–81	10.20	0.01	7.20	55–81	8.95	0.005
*QTKW2R.4*	84–87	86–89	10.68	0.01	7.60	86–89	4.81	0.05
*QTKW2R.5*		94–97	9.37	0.01	8.49	94–97	8.48	0.005
*QTKW3R.1*		23–29	6.17	0.05	5.29	23–26	6.90	0.01
*QTKW3R.2*	34–39	32–43	6.89	0.01	4.50	31–44	6.59	0.05
*QTKW3R.3*		47–49	4.74	0.05	4.50	46–50	5.60	0.05
*QTKW3R.4*	69–75							
*QTKW3R.5*		181–187	6.06	0.05	5.15	182–1897	5.01	0.05
*QTKW4R.1*	58–61	59–61	7.24	0.01	6.50	59–61	6.80	0.01
*QTKW5R.1*						15–19	8.30	0.005
*QTKW5R.2*	100–106	92–105	5.10	0.05	4.55	92–105	7.55	0.01
*QTKW5R.3*						111–115	4.56	0.05
*QTKW5R.4*	118–137					125–137	5.56	0.05
*QTKW5R.5*	145–156					139–145	5.56	0.05
*QTKW6R.1*		102–103	6.43	0.05	2.40	101–103	3.91	0.01
*QTKW6R.2*	108–115	107–115	6.60	0.05	5.07	108–115	6.69	0.05
*QTKW6R.3*		124–126	5.38	0.05	3.33	124–126	4.86	0.05
*QTKW6R.4*		184–187	6.77	0.05	4.00	184–187	4.39	0.05
*QTKW6R.5*		194–198	8.50	0.01	4.80	191–198	5.60	0.05
*QTKW6R.6*		208–210	4.55	0.05	4.00	209–211	4.90	0.05
*QTKW7R.1*	28–35	28–35	7.33	0.01	5.40	28–35	6.53	0.05
*QTKW7R.2*	53–56	54–56	7.58	0.01	4.60	52–56	5.24	0.05
*QTKW7R.3*		178	6.80	0.01	5.40	177–182	7.16	0.01
*QTKW7R.4*	195–199	194–200	9.93	0.01	7.05	187–200	9.15	0.005
*QTKW7R.5*	213–216	213–216	8.66	0.01	7.56	215–216	7.91	0.005

^a^Marker number on chromosome map

^b^LR, likelihood ratio test statistic compares two nested hypotheses (marker is linked to a QTL or not) and is two times the negative natural log of the ratio of the likelihoods (−2ln(L0/L1). Minimum significance at *p* < 0.05. The hypothesis H0: b1 = 0 to an alternative H1: b1 not 0 and that they have likelihoods L0 and L1 respectively

^c^*R*^2^ [%], the coefficient of determination: phenotypic variation explained by the marker in %

^d^The Kruskal–Wallis test statistic K* which measures the association between marker genotype and TKW segregation. For the *K* test, an association was indicated when the mean values of the marker classes were significantly different at *p* < 0.05

### Analysis of QTL interaction

A group of seven QTL was selected for analysis of two-loci interaction based on higher than 5% values of their difference between AA and BB genotypic values (Table 3). Genotypic values of AA genotypes in selected QTL exceeded the population mean by 2.9–4.7% and those of BB genotypes were lower than the population mean by 3.0–5.7% (Table 3). Differences between genotypic values of single QTL homozygotes AA and BB ranged from 6.4% for *QTKW3R.2* to 9.8% for *QTKW4R.1*. Genotypic difference between double homozygotes AA;AA and BB;BB varied from 9.9% (*QTKW7R.2-QTKW7R.5*) to 17.4% (*QTKW7R.5-QTKW6R.2*) (Tables 4, 5, 6, 7, and 8).

**Table 3:** Differences in genotypic values of AA (alleles from line 541) and BB (alleles from line Ot1–3) homozygotes for particular QTL controlling thousand-kernel weight in rye

QTL	Class of QTL revealed through analysis of population tails	Genotypic value of AA [%]	Genotypic deviation of AA from the population mean [%]	Genotypic value of BB [%]	Genotypic deviation of BB from the population mean [%]	Difference between genotypic values of AA and BB [%]
*QTKW4R.1*	R	65.0	+4.6	55.2	−5.2	9.8
*QTKW2R.3*	R	63.4	+3.0	54.7	−5.7	8.7
*QTKW2R.1*	E	65.1	+4.7	57.2	−3.2	7.9
*QTKW7R.2*	R	63.7	+3.3	55.9	−4.5	7.8
*QTKW7R.5*	D	64.5	+4.1	57.3	−3.1	7.2
*QTKW3R.2*	R	63.8	+3.4	57.4	−3.0	6.4
*QTKW6R.2*	R	63.3	+2.9	56.8	−3.6	6.5

**Table 4:** Distribution of genotypic values [%] for two-loci homozygotes and suggested interaction types between *QTKW2R.3* and other QTL for thousand-kernel weight. Genotypic differences lower than 4.5% threshold are in bold

QTL	*QTKW2R.3*	*QTKW2R.3*	Genotypic difference	Interaction type
Genotype	AA	BB		
*QTKW3R.2*	68.9	57.8	11.2	D-R
AA				
*QTKW3R.2*	62.2	55.6	6.6	
BB				
Genotypic difference	6.7	**2.2**	13.4	
*QTKW6R.2*	65.4	59.3	6.1	D-E
AA				
*QTKW6R.2*	61.5	52.3	9.2	
BB				
Genotypic difference	**3.9**	7.0	13.6	
*QTKW2R.1*	65.7	62.2	**3.5**	E*-E*
AA				
*QTKW2R.1*	61.6	54.6	6.5	
BB				
Genotypic difference	**4.1**	7.6	11.1	
*QTKW7R.2*	66.7	61.1	5.6	D-D
AA				
*QTKW7R.2*	59.1	54.5	4.6	
BB				
Genotypic difference	7.6	6.6	12.2

**Table 5:** Distribution of genotypic values [%] for two-loci homozygotes and suggested interaction types between *QTKW7R.5* and other QTL for thousand-kernel weight. Genotypic differences lower than 4.5% threshold are in bold

QTL	*QTKW7R.5*	*QTKW7R.5*	Genotypic difference	Interaction type
Genotype	AA	BB		
*QTKW2R.3*	67.6	60.5	7.1	D-D
AA				
*QTKW2R.3*	60.6	52.5	8.1	
BB				
Genotypic difference	7.0	8.0	15.1	
*QTKW2R.1*	66.4	61.1	5.3	D-E
AA				
*QTKW2R.1*	62.4	54.3	8.1	
BB				
Genotypic difference	**4.0**	6.8	12.1	
*QTKW3R.2*	65.0	58.9	6.1	R*-R*
AA				
*QTKW3R.2*	58.7	55.0	**3.7**	
BB				
Genotypic difference	6.3	**3.9**	10.0	
*QTKW6R.2*	68.8	60.7	8.1	D-D
AA				
*QTKW6R.2*	59.8	51.4	8.4	
BB				
Genotypic difference	9.0	9.3	17.4	
*QTKW7R.2*	64.7	61.4	**3.3**	E*-E*
AA				
*QTKW7R.2*	64.1	54.8	9.3	
BB				
Genotypic difference	**0.6**	6.6	9.9

**Table 6:** Distribution of genotypic values (%) for two-loci homozygotes and suggested interaction types between *QTKW6R.2* and other QTL for thousand-kernel weight. Genotypic differences lower than 4.5% threshold are in bold

QTL	*QTKW6R.2*	*QTKW6R.2*	Genotypic difference	Interaction type
Genotype	AA	BB		
*QTKW3R.2*	67.4	58.8	8.6	D-D
AA				
*QTKW3R.2*	59.9	53.7	6.2	
BB				
Genotypic difference	7.5	5.1	13.7	
*QTKW2R.1*	66.8	60.3	6.5	D-E
AA				
*QTKW2R.1*	62.6	51.3	11.3	
BB				
Genotypic difference	**4.2**	9.0	15.5	
*QTKW7R.2*	65.7	60.8	4.9	D-D
AA				
*QTKW7R.2*	59.8	54.7	5.1	
BB				
Genotypic difference	5.9	6.1	11.0

**Table 7:** Distribution of genotypic values (%) for two-loci homozygotes and suggested interaction types between *QTKW4R.1* and other QTL for thousand-kernel weight. Genotypic differences lower than 4.5% threshold are in bold

QTL	*QTKW4R.1*	*QTKW4R.1*	Genotypic difference	Interaction type
Genotype	AA	BB		
*QTKW2R.3*	67.0	55.2	11.8	D-R
AA				
*QTKW2R.3*	58.6	53.5	5.1	
BB				
Genotypic	8.4	**1.7**	13.5	
difference				
*QTKW7R.5*	68.7	57.2	11.5	D-R
AA				
*QTKW7R.5*	60.6	53.8	6.8	
BB				
Genotypic difference	8.1	**3.4**	14.9	
*QTKW3R.2*	67.1	57.2	9.9	D-R
AA				
*QTKW3R.2*	60.1	54.4	5.7	
BB				
Genotypic difference	7.0	**2.8**	12.7	
*QTKW2R.1*	67.7	59.4	8.3	D-D
AA				
*QTKW2R.1*	60.2	51.8	8.4	
BB				
Genotypic difference	7.5	7.6	15.9	
*QTKW6R.2*	66.0	58.4	7.6	D-D
AA				
*QTKW6R.2*	60.0	51.0	9.0	
BB				
Genotypic difference	6.0	7.4	15.0	
*QTKW7R.2*	66.6	60.1	6.5	D-D
AA				
*QTKW7R.2*	59.3	52.0	7.3	
BB				
Genotypic difference	7.3	8.1	14.6

**Table 8:** Distribution of genotypic values (%) for two-loci homozygotes and suggested interaction types between *QTKW3R.2*, *QTKW2R.1*, and *QTKW7R.2* for thousand-kernel weight. Genotypic differences lower than 4.5% threshold are in bold

QTL	*QTKW3R.2*	*QTKW3R.2*	Genotypic difference	Interaction type
Genotype	AA	BB		
*QTKW2R.1*	66.5	61.1	5.4	D-D
AA				
*QTKW2R.1*	59.3	53.7	5.6	
BB				
Genotypic difference	7.2	7.4	12.8	
*QTKW7R.2*	66.0	59.5	6.5	R*-R*
AA				
*QTKW7R.2*	58.3	55.7	**2.6**	
BB				
Genotypic difference	7.7	**3.8**	10.3	
	*QTKW7R.2*	*QTKW7R.2*		
	AA	BB		
*QTKW2R.1*	66.8	60.5	6.3	D-D
AA				
*QTKW2R.1*	61.3	55.2	6.1	
BB				
Genotypic difference	5.5	5.3	11.6	

**Fig. 3:**
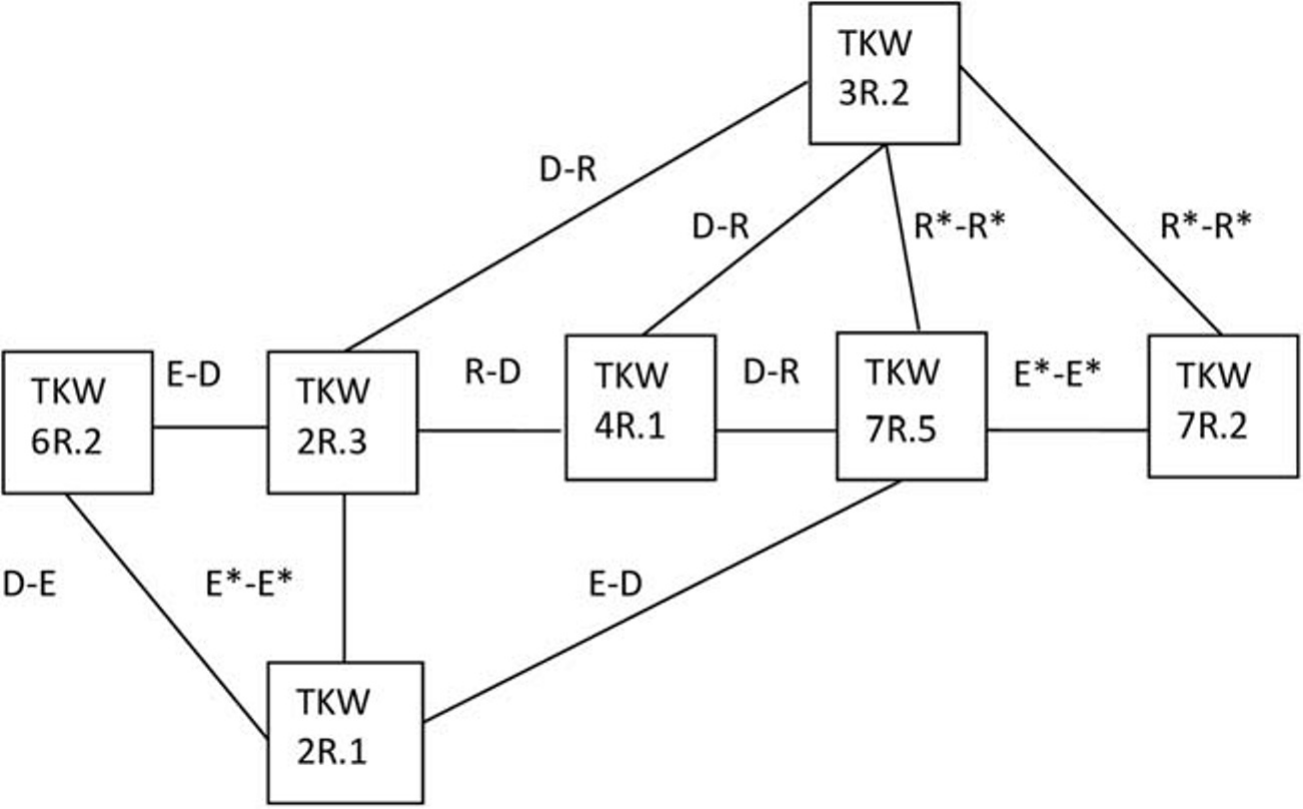
A network of 7 QTL for thousand-kernel weight (TKW) revealed through genetic analysis within the 541 × Ot1-3 mapping population of recombinant inbred lines of rye. Lines connecting QTL reflect their possible interaction classified according to Masojć et al. (2016)

Distributions of genotypic values for two-loci homozygous genotypes representing particular pairs of QTL (Tables 4, 5, 6, 7, and 8, Fig. 3) corresponded to those described in the model of two-loci interactions (Masojć et al. 2016). Individual QTL were involved in various types of two-loci interactions. Ten pairs of QTL (*QTKW2R.3-QTKW7R.5*, *QTKW2R.3-QTKW7R.2*, *QTKW6R.2-QTKW7R.5*, *QTKW3R.2-QTKW6R.2*, *QTKW7R.2-QTKW6R.2*, *QTKW4R.1-QTKW6R.2*, *QTKW4R.1-QTKW2R.1*, *QTKW4R.1-QTKW7R.2*, *QTKW3R.2-QTKW2R.1*, and *QTKW7R.2-QTKW2R.1*) showed an additive relationship of the D-D type. Epistatic interaction of the D-R type can be suggested for four QTL pairs (*QTKW2R.3-QTKW3R.2*, *QTKW4R.1-QTKW2R.3*, *QTKW4R.1-QTKW7R.5*, and *QTKW4R.1-QTKW3R.2*). The scheme characteristic for D-E type of epistatic interaction is seen in the case of three QTL pairs (*QTKW6R.2-QTKW2R.1*, *QTKW7R.5-QTKW2R.1*, and *QTKW2R.3-QTKW6R.2*). Complementary interaction of E*-E* type is revealed by two QTL pairs (*QTKW2R.3-QTKW2R.1* and *QTKW7R.5-QTKW7R.2*). Complementary interaction of R*-R* type can be assigned to two QTL pairs (*QTKW7R.5-QTKW3R.2* and *QTKW7R.2-QTKW3R.2*).

*QTKW3R.2* was characterized by GIABDS as an R class locus and expressed an epistatic R-D type of interaction with *QTKW2R.3* and *QTKW4R.1* and complementary R*-R* type of interaction with *QTKW7R.5* and *QTKW7R.2*. It can be concluded that the R class of *QTKW3R.2* complies with its profile of two-loci interactions. Confirmation of the QTL classification gained through GIABDS analysis was also obtained for *QTKW2R.1* of class E as it was involved in E-D type of interaction with *QTKW6R.2* and *QTKW7R.5* and in E*-E* type of interaction with *QTKW2R.3*. *QTKW7R.5* interacted with other QTL on a variety of ways (D-E, R-D, R*-R* and E*-E*). Allele B at this QTL was significant in TKW reduction through cumulative action with B alleles at *QTKW2R.1* and *QTKW7R.2* loci, while allele A positively affected TKW in combination with A alleles at *QTKW4R.1* and *QTKW3R.2* loci. These observations suggest that A and B alleles at the *QTKW7R.5* locus were significant for positive and negative directions of TKW selection, respectively. Thus, the interactions profile of *QTKW7R.5* seems to correspond to its class D revealed through analysis of alleles distribution within-population tails (Table 1). *QTKW4R.1* was found to be involved only in D-R type of interactions with three other loci, and therefore, it played an important role in the cumulative effect of alleles A, elevating TKW value. Such interaction profiles for this QTL seems to comply with its classification as the R class locus (Table 1). *QTKW7R.2* confirmed its classification (R* class) in relation with *QTKW3R.2* (R*-R* type of interaction) but not in relation with *QTKW7R.5* (E*-E* type of interaction). *QTKW2R.3* was detected as a class R locus (Table 1) and revealed similar status in relation to *QTKW4R.1* and the same type of relationship with *QTKW3R.2*. It was also involved in different types of interaction with *QTKW6R.2* (D-E type) and *QTKW2R.1* (E*-E* type). *QTKW6R.2* of class R was mainly involved in an additive relationship of D-D type with other QTL. Interactions of this QTL with *QTKW2R.1* (D-E type) and with *QTKW2R.3* (E-D type) seem to be incompatible with its status revealed within population tails.

## Discussion

A system of 28 QTL for TKW was revealed in the 541 × Ot1-3 mapping population of RILs using three methods of QTL detection. This result confirms earlier reports showing the complexity of genetic control of the crucial yield component in rye (Miedaner et al. 2012; Falke et al. 2009; Myśków et al. 2014; Hackauf et al. 2017). Some apparent similarities in QTL location can be found in parallel studies. It can be noted for *QTKW7R.5* (this paper) and QTL from the distal region of chromosome arm 7RL reported by Miedaner et al. (2012) in two mapping populations. Both studies also disclose QTL for TKW on the short arm of chromosome 3R. Colocation of QTL from chromosomes 2RL, 3R, and 4R reported by Hackauf et al. (2017) and *QTKW2R.3*, *QTKW3R.2*, and *QTKW4R.1* characterized in the present study should also be acknowledged. Confirmation of effective QTL on chromosomes 2R and 4R in the two independent investigations are especially valuable. A similar map location to that occupied by *QTKW6R.2* (i.e., the long arm of chromosome 6R), was also revealed for QTL detected in mapping population explored by Myśków et al. (2014). Colocation of QTL for TKW in two, not related mapping populations was observed on chromosome 2RL (*QTKW2R.3*) (Milczarski and Masojć 2003 and the present study). However, numerous QTL for TKW observed in various bidirectional populations possibly represent different loci since the distribution of DNA polymorphisms is often independent in not related plant materials.

Confronting GIABDS, a relatively new method of QTL detection (Masojć et al. 2009, 2011, 2016, 2017), with the classic approach in QTL mapping represented by SMA and K-W methods confirmed the reliability of the first method. Thirteen QTL for TKW revealed by GIABDS coincided with QTL determined by the two remaining methods. SMA and K-W proved to be more sensitive in QTL detection than GIABDS since they revealed an additional 14 QTL. It can be explained by a larger set of data available for the two classic methods as they are based on the analysis of the overall mapping population (144 RILs) while GIABDS was performed on data from two selected sets of 24–25 RILs each, representing population tails. Nevertheless, GIABDS analysis delivers unique information about the QTL class, which informs whether a given QTL is important for selection aimed at improving agronomic trait. It shows that only D, R, and R* QTL classes represent positive allele-trait associations while the remaining classes as E or E* are not valuable for selection purposes (Masojć et al. 2009, 2016). GIABDS analysis also offers a new approach for assessing QTL interaction.

A growing number of studies are revealing the interactions between genes or QTL for such characteristics as heading date (Tranquilli and Dubcovsky 2000), falling number (Deng et al. 2014), and developmental traits (Wang et al. 2010) in wheat, yield-related traits (Xing et al. 2002) and male sterility (Long et al. 2008) in rice, or developmental traits in *Arabidopsis* (Smith and Hake 2003; Aida et al. 1999). A complementary interaction between two loci for TKW located on chromosomes 5R and 7R in the rye was suggested by Wricke (2002). Results of QTL mapping for TKW in the 541 × Ot1-3 mapping population, strongly support the hypothesis about the important role of genes interaction in controlling the weight of rye grain.

The present study is a first attempt to check the new model of two-loci interactions based on the analysis of subpopulations with extreme trait values (Masojć et al. 2016). It was tested on seven QTL detected by both QTL mapping using the overall population and GIABDS methods. This fact adds TKW to two other traits of rye (PHS, AA) where the validity of QTL detection by GIABDS method has been proven by the classic approach (Masojć et al. 2009, 2011).

Differences between genotypic values of double homozygotes most frequently revealed independence of allelic effects at one QTL from genotype at the second QTL, which was characteristic for the additive relationship of the D-D type. However, in several tested two-loci combinations, one or two differences between genotypic values were below a threshold level, which suggested interaction. Four types of two-loci interaction consistent with the model schemes were postulated, such as the epistatic interaction of the D-R and D-E types and complementary interaction of the E*-E* and R*-R* types. The model assumed that the class of QTL revealed by analysis of AA and BB frequencies within subpopulations of extreme trait values results from two-loci interactions and, therefore, should be correlated with a specific distribution of genotypic values of four double homozygotes. Such confirmation of the QTL classification was gained for *QTKW3R.2* (R class locus) and *QTKW2R.1* (E class locus). Class D of *QTKW7R.5* can also be confirmed by a specific combination of interactions with other loci, important for both negative (D-E, E*-E*) and positive (R*-R*, R-D) direction of selection for TKW. Class R expressed by *QTKW4R.1* seems to be consistent with its D-R type of interactions with three QTL, possibly leading to the high value of its A allele for positive direction of selection and weak effects for the opposite direction. It is difficult to ascertain the class of remaining QTL from comparing genotypic values in two-loci schemes since they showed various types of possible interactions. It seems that QTL class obtained through analysis of subpopulations with extreme trait values should be considered as an outcome of predominant two-loci interactions, characterizing the status of specific QTL in a complex network of interactions. The main value of these QTL characteristics is a suggestion about the significance of particular alleles for selection in positive (classes R or R*), negative (classes E or E*), or in both (class D) directions, relative to breeding value. By confronting the theoretical model presented earlier (Masojć et al. 2016) with experimental data for TKW in the rye, a high level of consistency has been found. Bear in mind that in the model examples only two loci and not a multi-loci network controlling quantitative trait were considered.

It seems that the D-D type of relationship between two QTL does not always indicate their high rank. Such a relationship exists between the most important QTL for TKW (i.e., *QTKW2R.3* and *QTKW7R.5*, *QTKW4R.1*, and *QTKW7R.2*) but also between QTL of lower rank (e.g., *QTKW2R.1* and *QTKW3R.2*). It seems likely that the D-D relationship informs merely about the additive effects of the two-loci genotypes. The superior role of QTL results from its involvement in several epistatic and/or complementary interactions increasing control over the quantitative trait. Therefore, *QTKW4R.1*, *QTKW7R.5*, and *QTKW2R.3* should be considered as key QTL in controlling high TKW within the 541 × Ot1-3 mapping population.

Pyramiding of A or B alleles in two or three interacting QTL showed that the extreme values of TKW could be reached by combining the proper alleles at four or five QTL. It seems that a subpopulation of RILs with extreme phenotypes contains several different combinations of the selected four or five loci genotypes with positively acting alleles. This hypothesis reveals several characteristics of the GIABDS analysis. First, it is understandable why heterogeneity (overrepresentation of one genotype) and not homogeneity (100% frequency of one allele) is observed at a particular QTL within the selected subpopulations of extreme trait values. This is because the highest trait values can be achieved by pyramiding alleles at different sets of four or five QTL. Since a number of effective QTLs are usually higher than four or five, the lack of a proper allele from one QTL can be compensated by the presence of effective alleles in another QTL. This feature of the GIABDS analysis increases the sensitivity of the QTL detection because two or three loci genotypes can attain highly differentiated genotypic values and might be efficiently separated through divergent selection. This mechanism allows the recognition of numerous QTLs with not high allelic effects.

The QTLs selected in this way, which are the most important for traits formation, become the basis for the selection of genotypes with the required properties. To this end, a set of allele-specific markers strongly associated with the trait should be prepared, for crossbreeding that will guarantee an optimal effect. The method applied allows the reduction of the number of QTLs considered for the selection process only to those that are positively targeted.

In conclusion, a phenotypically highly differentiated bi-parental population consisting of 120–150 RILs can be used for GIABDS analysis. Populations of this size allow us to select the two opposite subpopulations with extreme trait values consisting of c. 20 lines each, which is an acceptable number for testing the significance of segregation distortion. Validation of QTL detected through GIABDS analysis by classic QTL mapping support the notion that this new method is useful in the characterization of QTLs within populations of RILs. GIABDS may also be applied for the analysis of two-loci interactions according to the genetic model developed earlier.

## Electronic supplementary material

ESM 1 (XLSX 344 kb)
